# Use of semantic workflows to enhance transparency and reproducibility in clinical omics

**DOI:** 10.1186/s13073-015-0202-y

**Published:** 2015-07-25

**Authors:** Christina L. Zheng, Varun Ratnakar, Yolanda Gil, Shannon K. McWeeney

**Affiliations:** Division of Bioinformatics and Computational Biology, Department of Medical Informatics and Clinical Epidemiology, Oregon Health & Science University, Portland, OR USA; Knight Cancer Institute, Oregon Health & Science University, Portland, OR USA; Information Sciences Institute, University of Southern California, Los Angeles, CA USA; Division of Biostatistics, Department of Public Health and Preventative Medicine, Oregon Health & Science University, Portland, OR USA

## Abstract

**Background:**

Recent highly publicized cases of premature patient assignment into clinical trials, resulting from non-reproducible omics analyses, have prompted many to call for a more thorough examination of translational omics and highlighted the critical need for transparency and reproducibility to ensure patient safety. The use of workflow platforms such as Galaxy and Taverna have greatly enhanced the use, transparency and reproducibility of omics analysis pipelines in the research domain and would be an invaluable tool in a clinical setting. However, the use of these workflow platforms requires deep domain expertise that, particularly within the multi-disciplinary fields of translational and clinical omics, may not always be present in a clinical setting. This lack of domain expertise may put patient safety at risk and make these workflow platforms difficult to operationalize in a clinical setting. In contrast, semantic workflows are a different class of workflow platform where resultant workflow runs are transparent, reproducible, and semantically validated. Through semantic enforcement of all datasets, analyses and user-defined rules/constraints, users are guided through each workflow run, enhancing analytical validity and patient safety.

**Methods:**

To evaluate the effectiveness of semantic workflows within translational and clinical omics, we have implemented a clinical omics pipeline for annotating DNA sequence variants identified through next generation sequencing using the Workflow Instance Generation and Specialization (WINGS) semantic workflow platform.

**Results:**

We found that the implementation and execution of our clinical omics pipeline in a semantic workflow helped us to meet the requirements for enhanced transparency, reproducibility and analytical validity recommended for clinical omics. We further found that many features of the WINGS platform were particularly primed to help support the critical needs of clinical omics analyses.

**Conclusions:**

This is the first implementation and execution of a clinical omics pipeline using semantic workflows. Evaluation of this implementation provides guidance for their use in both translational and clinical settings.

**Electronic supplementary material:**

The online version of this article (doi:10.1186/s13073-015-0202-y) contains supplementary material, which is available to authorized users.

## Background

High throughput ‘omics’ technologies such as genomics, proteomics, metabolomics, etc. hold great promise for precision medicine wherein a patient’s personal omics data are used to inform individualized care. Recently published preclinical omics studies highlight the tremendous potential omics can have on improving patient care through assessing disease risk [1–4], averting potential adverse drug reactions [5–7], and ultimately tailoring treatment to the individual, not the disease [8–10]. The potential of having disease traits be monitored through omics data of healthy individuals [11] has also garnered much excitement.

Despite the large number of published preclinical omics studies, only a few have been successfully translated into a clinical setting [12, 13]. The primary scientific causes for this have been attributed to 1) preclinical omics studies not being adequately designed to answer the intended clinical question and 2) inadequate statistical or bioinformatics rigor [14]. The latter issue has garnered much attention with respect to both the benchmarking and quality control of omics analysis pipelines and the transparency and reproducibility of those pipelines once they are established. Efforts to benchmark the accuracy, biases, and sources of errors within omics analysis methods are critical to translational and clinical omics [15]. On the heels of the US Food and Drug Administration’s (FDA) approval of the first next-generation sequencing instrument [16], their recent public workshop on next generation sequencing standards highlighted the critical need for the quality assurance of computational biology pipelines [17]. Towards these efforts, the National Institute of Standards and Technology (NIST), in conjunction with the Genome in a Bottle Consortium, recently published a set of high-confidence, genome-wide single-nucleotide polymorphism (SNP), indel and genotype calls, based on a genome sequence that they have established as a DNA reference material and made freely available to be used as a truth table in the benchmarking of bioinformatics methods for identifying DNA variants from sequenced genomes [15]. Unfortunately, efforts towards making clinical omics analysis pipelines more transparent and reproducible are still in their infancy. Even in the clinical and translational research domain, there has been a critical need for computational transparency and reproducibility [14, 18]. This is exemplified by a recent study in which over 1500 person hours were dedicated to the ‘forensic omics’ task of deciphering the exact data sets used and determining how the data were processed for assignment of patients to clinical trials [19].

Thus, a key challenge now is how we can increase transparency and reproducibility. This question is pertinent to clinical omics and the scientific community as a whole [20–22]. This is highlighted by the recent work of Garijo et al. [23], whose efforts to reproduce a published computational method led them to publish a set of reproducibility guidelines for authors. They recommend that authors include all pertinent data: the exact input data used, key intermediate data, output data, and any third party data (i.e., from external databases) for the analysis. They also recommend the inclusion of all software code, parameters, and configuration files necessary for the analysis. Finally, they recommended including a high level flow diagram to guide users through the entire approach. Two recent reports echoed similar requirements for translational and clinical omics with the addition of key transparency requirements, including the need for data provenance to help ensure data integrity and the need to enhance analytical validity to help ensure “we are doing the test correctly” [14, 18]. We have summarized the requirements across these studies into a checklist to facilitate the evaluation of transparency and reproducibility in translational and clinical omics (Table 1).

**Table 1:** Criteria checklist for enhanced transparency and reproducibility in clinical omics

▪ Exact input data used for the analysis
▪ Key intermediate data generated from the analysis
▪ Third party data (i.e., data from external sources)
▪ Output data
▪ Provenance of all data used
▪ All code/software used in the analysis
▪ Provenance of all code used
▪ Documentation of computing environment used
▪ Veracity checks to ensure analytical validity
▪ High-level flow diagram describing the analysis

Workflow systems such as Galaxy [24] and Taverna [25] help to meet many of the requirements listed above and have greatly enhanced the use, transparency, and reproducibility of omics pipelines in the research domain [25, 26]. With these systems, exact input, key intermediate, final output, and relevant external data are all preserved. All code, computational configurations, parameters, and their provenance can be captured within these systems. These systems also provide a high level flow diagram to guide users through execution. However, a key requirement is inherently missing from these systems: there is no way to include veracity checks during workflow runs to enhance analytical validity. The execution of workflows within these systems therefore requires deep domain knowledge and expertise to ensure data integrity and analytical validity. For example, it is the user’s responsibility to ensure that the correct input is provided; the systems do not inherently validate the input provided, nor do they provide guidance to the user of the appropriate input needed. Particularly within multi-disciplinary fields such as translational and clinical omics where expertise from clinicians, laboratory personnel, bioinformaticists, and statisticians must be effectively integrated and navigated, expertise across all fields may not always be present in ‘real time’ in the clinical setting, thus putting patient safety at risk and making these workflow platforms inadequate for a clinical setting.

We recently investigated the use of semantic workflows with the analysis of multi-omics data and found that the encapsulation of multi-step omics analysis methods within a semantic framework resulted in a transparent, reproducible, and semantically validated analysis framework [27], making semantic workflows a potential viable candidate for clinical omics. Semantic workflows are a unique and different class of workflow platforms. Similar to other workflow systems, semantic workflows manage and record the execution of complex computations, record provenance, and allow end-users to reproduce workflows. However, unique to semantic workflow systems is their ability to generate semantically validated workflow runs wherein domain expertise can be encoded within user-defined rules and constraints, and these rules and constraints are semantically enforced to help guide users through a workflow run. This guidance enhances data integrity and analytical validity throughout a workflow run, thus making semantic workflows a potential candidate for meeting the critical needs of transparency, reproducibility, and analytical validity in a clinical setting.

To evaluate the use of semantic workflows within clinical omics, we have implemented and executed the first clinical omics analysis pipeline using the Workflow Instance Generation and Specialization (WINGS) semantic workflow platform [28]. We found the WINGS platform capable of effectively meeting the checklist of requirements for enhanced transparency, reproducibility, and analytical validity recommended for translational and clinical omics defined at the beginning of this study. We further found that many features of the WINGS platform were particularly effective in supporting the critical needs of clinical omics analyses, such as the need to keep pace with frequent updates of biological life science databases, to enforce consistency/data integrity across heterogeneous biological/clinical data, to keep pace with rapid updates/development of omics software tools, and to process large omics data sets.

## Methods and results

### Use-case: clinical omics analysis pipeline

The clinical omics pipeline use-case, in this study, is a DNA variant annotation pipeline, provided by the Knight Diagnostic Laboratories (KDL) at Oregon Health and Science University (OHSU) for this implementation, aimed at coalescing molecular, pathogenic, and population annotation information on DNA variants identified through DNA sequencing from a patient’s tumor sample. DNA sequencing was performed on the Ion Torrent Personal Genome Machine (PGM^™^) System for Next-Generation Sequencing, using the GeneTrails Solid Tumor Panel®, which delivers information on 37 genes commonly involved in solid tumors.

The omics annotation pipeline begins with a file of sequenced DNA variants from a patient’s tumor sample. All identified DNA sequence variants are annotated with the following information: 1) potential effect on the resultant protein(s); 2) annotation within the Catalogue of Somatic Mutations in Cancer (COSMIC) database [29]; and 3) annotation within the Single Nucleotide Polymorphism database (dbSNP) [30]. The potential molecular effect of the DNA variant on the amino acid sequence of the resultant protein(s) (e.g., non-synonymous) is analyzed using the Bioconductor VariantAnnotation package [31]. Information regarding the DNA variants' potential pathogenic associations with cancer and their frequency within the population is obtained through COSMIC and dbSNP, respectively. Additional manually curated information regarding the DNA variants (e.g., if it is within a homo-polymer region), if available, is also incorporated. The final output of the annotation pipeline is a file coalescing all of the obtained annotation information for all identified DNA variants from the patient’s tumor sample. This output is then used by clinicians to aid in determining individualized patient care.

This DNA variant annotation pipeline use-case involves a small number of annotation resources; however, even at this level, the importance of and difficulty in adhering to the requirements of transparency, reproducibility and accuracy is evident. For example, the computational code for this analysis pipeline was stored on multiple desktop machines and executed by multiple laboratory personnel. The lack of a central location for the storage and execution of the code exposed opportunities for potential errors and inconsistencies, making reproducibility very difficult. The use of multiple workstations introduced potential inconsistencies arising from the use of different versions of software or code. Potential errors or inconsistencies might have also arisen from unmet constraints such as ensuring that all genomic coordinates among the different annotation resources are of the same genomic assembly. Additionally, a lack of version control and automated provenance tracking of the annotation sources further complicates the task of accuracy and reproducibility.

### The WINGS semantic workflow system

The WINGS workflow system [28] is a unique class of workflow platforms wherein analysis pipelines are transformed into transparent, reproducible, semantically validated workflow runs. Similarly to other workflow systems, through the encapsulation of analysis steps into individual workflow components with predefined inputs, outputs, and parameters, WINGS tracks and records the provenance of complex computations and enables end-users to reproduce workflows. However, unique to WINGS is its ability to generate semantically validated workflow runs wherein all components and datasets are automatically checked for coherence and consistency and all user-defined rules and constraints are semantically enforced. WINGS accomplishes this through two features not found in other workflow platforms: 1) the integration of individual workflow components and their datasets; and 2) the semantic enforcement of user-defined rules and constraints. Formal descriptions and detailed algorithms for WINGS can be found in Gil et al. [32].

The integration of individual workflow components and their datasets within WINGS is achieved through the use of individual ontologies used to define and organize all datasets and workflow components, respectively. Within the dataset ontology, categories are defined for each dataset, and within the workflow component ontology, categories are defined for each workflow component. Categories can be developed using study custom or standardized biological ontologies (e.g., EDAM [33], SeqOntology [34, 35], etc.). In this way, all datasets and workflow components are clearly defined (e.g., metadata, parameters) and organized within their individual categories. These categories can then be used to define relationships within an individual ontology such as defining one dataset as a subclass of an existing dataset or defining one workflow component as a subclass of an existing workflow component. These categories can also be used to define relationships across the two ontologies, such that the use of specific dataset categories can be restricted or pre-set within individual workflow components. The ability for cross-talk between the two ontologies creates an unprecedented integration between workflow components and their datasets wherein only predefined datasets are used and set throughout the workflow, thus maintaining data integrity. Within other workflow platforms, such as Galaxy and Taverna, which do not have this level of integration, data integrity is at risk, as the correct usage of datasets throughout a workflow run is not automatically verified. Although Galaxy and Taverna workflow components can explicitly be defined to specify the format type (e.g., FASTA file, SAM/BAM format) of required datasets, no explicit inherent format type checking is performed to ensure that a dataset of the specified format type was provided by the user.

Further enhancing the ability of WINGS to create semantically validated workflow runs is that it can semantically enforce user-defined rules and constraints. In doing so, workflow developers are able to further refine relationships across and between datasets and workflow components. For example, developers can constrain all datasets within a workflow run to have a specific metadata value (for instance, specific genome assembly). Rules can also be defined to require that specific datasets be processed by specific workflow components (described further below). In essence, through the use of predefined rules and constraints, domain knowledge and expertise is embodied and disseminated with each workflow. This not only enhances the analytical accuracy and validity of each workflow run, but it also guides users through a workflow run as error messages are displayed if any rule or constraint is violated. Optional semantically validated datasets can also be suggested upon user request.

WINGS has other functionality that is not directly related to its semantic capabilities [36]. One is the large-scale execution of workflows, which was one of the first capabilities incorporated in WINGS to support large-scale earthquake simulations [37]. Once a workflow is set up, WINGS can execute it in several alternative modes [38]. In one mode, its execution environment can be a local host, with WINGS generating scripted codes, or a distributed execution on a network of local machines. Alternatively, WINGS can generate execution-ready workflows that can be submitted to either Apache OODT [39] or the Pegasus/Condor execution engine [40], which are designed for large-scale distributed data processing in a variety of environments, such as local clusters, shared infrastructure, or cloud resources. Furthermore, based on user-defined execution requirements, WINGS can automatically generate the most appropriate and/or efficient workflows [41]. WINGS has not, however, been used to compose web services into workflows while other workflow systems such as Taverna can support it.

WINGS publishes and shares workflows using the W3C PROV-O ontology for workflow executions and its extension OPMW to represent workflow templates [42, 43]. OPMW is based on the W3C PROV model as well as the earlier Open Provenance Model adopted by many workflow systems [44]. OPMW supports the representations of workflows at a fine granularity with a lot of details pertaining to workflows that are not covered in more generic provenance models [45]. OPMW also allows the representation of links between a workflow template, a workflow instance created from it, and a workflow execution that resulted from an instance. Finally, OPMW also supports the representation of attribution metadata about a workflow, which some applications consume.

The WINGS workflow repository is publicly available and is part of the WEST ecosystem [46] that integrates different workflow tools with diverse functions (workflow design, validation, execution, visualization, browsing and mining) created by a variety of research groups. These tools include LONI Pipeline [47], Apache OODT and Pegasus/Condor. The workflow repository has been used to mine workflow patterns [46, 48]. WEST uses workflow representation standards and semantic technologies to enable each tool to import workflow templates and executions in the format they need. WEST is the first integrated environment where a variety of workflow systems and functions interoperate, and where workflows produced by a given tool can be used by more than one other tool. Other benefits of this approach include the interoperability among the applications in the ecosystem, the flexibility to interchange data, and facilitating the integration of content modeled in other vocabularies. Our representations are mapped to an extension of PROV for reusable plans called P-PLAN [49] as a basis to further map to processes other than workflows such as scientific experiments that use ISA [50]. Workflow repositories such as myExperiment [51] and CrowdLabs [52] can be used for sharing scientific workflows created with other systems. These workflows are reused by scientists that seek, retrieve, and reapply them. However, these workflows are not described with any structured annotations or constraints that capture their applicability as WINGS does.

Other workflow systems used in biomedical research such as LONI Pipeline, Taverna, GenePattern [53], and Galaxy offer very useful capabilities, and include libraries of components that are widely used in the community, such as genomic analysis tools or Bioconductor services [54]. However, their workflow representations specify the software to run at each step, but do not represent constraints such as whether an algorithm is appropriate given a dataset’s characteristics or how to set a software tool’s parameters to get best results. The SADI framework proposes best practices for documenting services with semantic constraints, and provides a plug-in for Taverna where services can be incorporated into the workflow based on semantic constraints, but does not support constraint propagation and reasoning at the workflow level [55]. WINGS is unique in capturing such semantic constraints. Please refer to Additional file 1 for additional information on the WINGS system.

### Implementation of a clinical omics workflow using the WINGS semantic workflow system

The first step in implementing a WINGS semantic workflow is for a workflow developer to create all datasets, components, rules, and constraints needed for an analysis pipeline. These are then used to build the workflow template needed for workflow users to execute reproducible and semantically validated workflow runs. Each is described in more detail below.

#### Datasets and their metadata

**Fig. 1:**
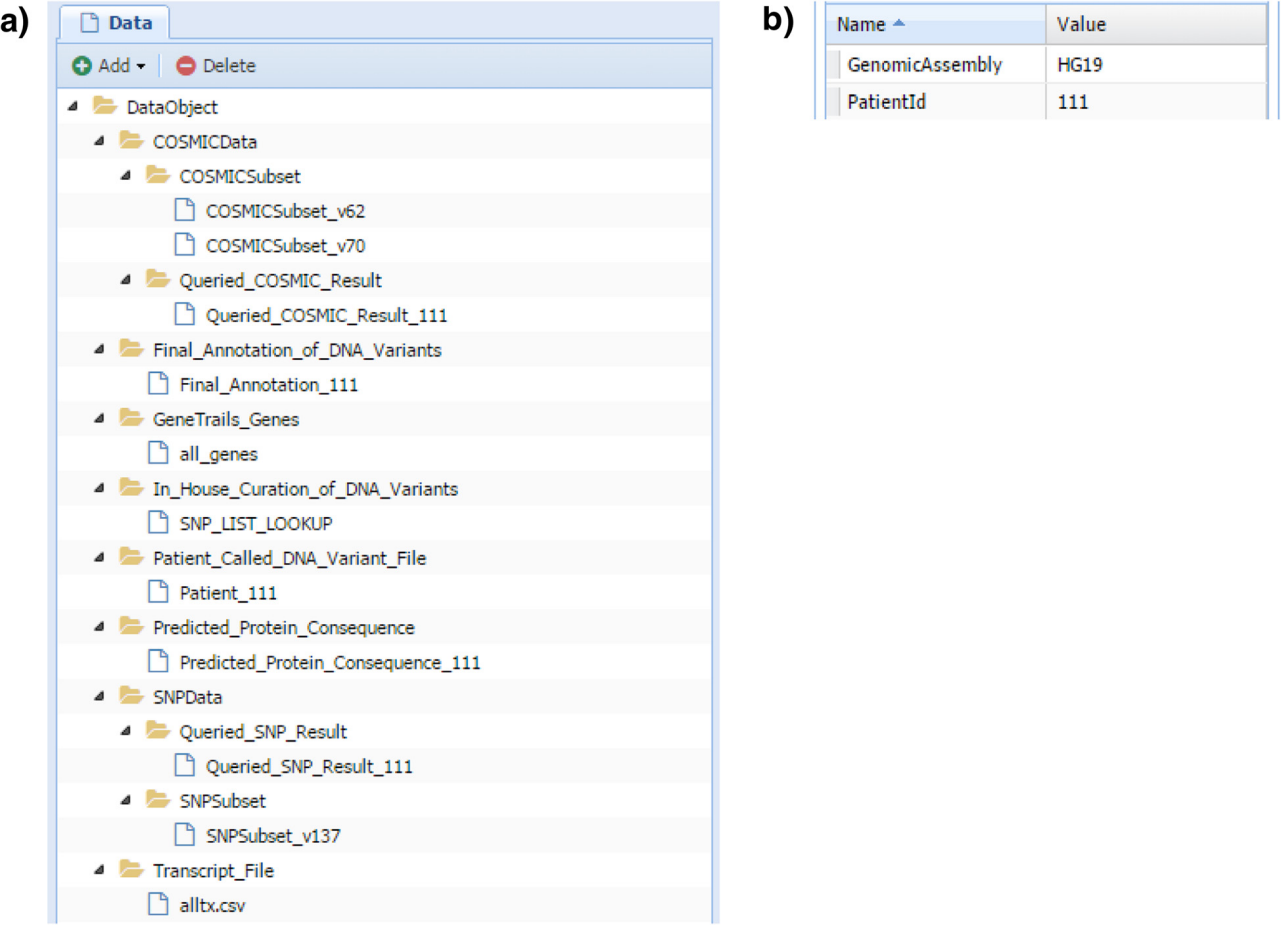
WINGS datasets ontology for our clinical omics use-case. WINGS datasets — any input, output, or intermediate data files — within a workflow template are classified within an ontology. (**a**) The ontology classifying the datasets within our WINGS omics workflow is shown. Each dataset can be defined as an individual class or defined as a subclass of an existing dataset. Patient_Called_DNA_Variant_File is an example of an individually defined dataset class while COSMICSubset and Queried_COSMIC_Result are examples of subclasses under the COSMICData dataset. Each dataset can be further defined with metadata. (**b**) The defined metadata and its value for a Patient_Called_DNA_Variant_File are shown

Datasets consist of any input, output, or intermediate data files within an analysis pipeline. For example, within our DNA variant annotation pipeline, key datasets include 1) Patient_Called_DNA_Variant_File, the file of sequenced DNA variants from a patient’s tumor; 2) COSMICSubset, the GeneTrails-specific subset of COSMIC; 3) SNPSubset, the GeneTrails-specific subset of dbSNP; and 4) Final_Annotation_of_DNA_Variants, the final annotation file of the identified DNA variants. Please refer to Table 2 for a complete list of datasets found within our pipeline. Because all datasets are defined within an ontology, WINGS is able to effectively organize and constrain the use of each dataset (Fig. 1a). We note that custom or standardized ontologies (e.g., the Sequence Ontology which not only represents the DNA variants but also contains the Protein Feature Ontology to handle protein consequence [56]) can easily be used. Some datasets are defined as their own entity (e.g., GeneTrails_Genes or Patient_Called_DNA_Variant_File) while others are defined as subclasses to other datasets (e.g., Queried_SNP_Result and SNPSubset are subclasses of SNPData). By defining datasets as subclasses to other datasets, common metadata can be shared among the parent and child datasets. For example, dbSNPVersionId is common metadata for SNPData, SNPSubset, and Queried_SNP_Result datasets. Metadata for each dataset can be defined, populated, updated, and viewed using the WINGS framework (Fig. 1b). Metadata can also be automatically populated and propagated throughout a workflow run. For a complete list of metadata used in our workflow, please refer to Additional file 1.

**Table 2:** WINGS datasets for our clinical omics use-case

Dataset	Description
GeneTrails_Genes	List of genes on the GeneTrails Solid Tumor Panel®
COSMICSubset	GeneTrails specific subset of COSMIC
SNPSubset	GeneTrails specific subset of dbSNP
Patient_Called_DNA_Variant_File	Identified DNA variants from a patient’s tumor sample
Queried_COSMIC_Result	Queried COSMIC annotation specific to a Patient_Called_DNA_Variant_File
Queried_SNP_Result	Queried dbSNP annotation specific to a Patient_Called_DNA_Variant_File
Transcript_File	Transcripts of interest from GeneTrails_Genes
Predicted_Protein_Consequence	Predicted consequence(s) specific to a Patient_Called_DNA_Variant_File
In_House_Curation_of_DNA_Variants	Manually curated information on sequence characteristics of previously identified DNA variants
Final_Annotation_of_DNA_Variants	Coalesced annotation information from the workflow specific to a Patient_Called_DNA_Variant_File

#### Workflow components

Workflow components define and encapsulate each step of an analysis pipeline. Similarly to datasets, all WINGS components are classified using an ontology where an individual component can either be classified as its own entity or grouped under a super-component class termed “component-type”. Component-types are used to group components sharing a common base set of input/output datasets such as those encapsulating code for different versions of the same tool or different tools performing similar functions. Component-types can also be used to effectively organize and enhance the flexibility of individual components within a workflow template wherein components can be easily incorporated into existing component-types with their use semantically enforced (discussed further below).

**Fig. 2:**
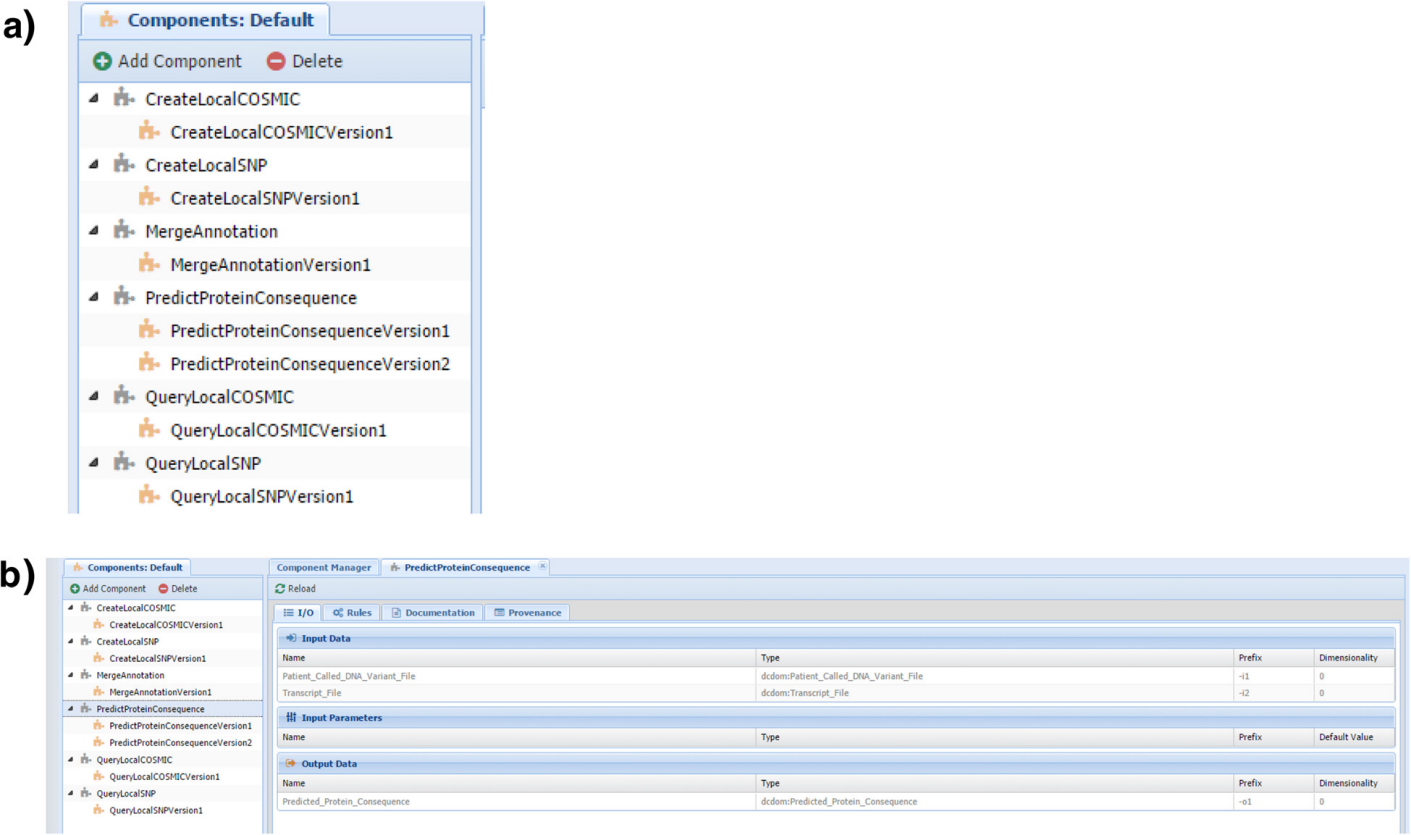
WINGS workflow components ontology for our clinical omics use-case. WINGS components are used to encapsulate individual steps of an analysis pipeline and are classified within an ontology in a workflow template. Individual components can be classified as their own component-class or as a subclass of a component-type. Component-types are used to group components sharing a common base set of input and output datasets such as those encapsulating code for different versions of the same tool or different tools performing similar functions. Component-types can also be used to effectively organize and enhance the flexibility of individual components within a workflow template. Each step of our clinical omics analysis pipeline was encapsulated within a component-type, even if only one component is currently defined (**a**). Individual component-types are shown in *grey* while individual components are depicted in *yellow*. Each component is defined with the following: 1) input datasets, 2) computational code, and 3) output datasets. For example, each *PredictProteinConsequence* component was defined with the following two input datasets: 1) Patient_Called_DNA_Variant_File and 2) Transcript_File and the following output dataset: 1) Predicted_Protein_Consequence (**b**). The R code needed for the analysis of this step was included to complete the creation of the component

To capitalize on the many features of component-types, each step of our clinical omics pipeline was segregated into the following component-types: 1) *CreateLocalCOSMIC*, 2) *CreateLocalSNP*, 3) *QueryLocalCOSMIC*, 4) *QueryLocalSNP*, 5) *PredictProteinConsequence*, and 6) *MergeAnnotation* (Fig. 2a). *CreateLocalCOSMIC* created a dataset containing a subset of COSMIC annotation specific for genes found on the GeneTrails Solid Tumor Panel®. *CreateLocalSNP* creates a dataset containing a subset of dbSNP annotation specific for genes found on the GeneTrails Solid Tumor Panel®. *QueryLocalCOSMIC* queried the COSMIC subset dataset for annotation information pertaining to a file of identified DNA variants from a patient’s tumor sample. *QueryLocalSNP* queried the dbSNP subset dataset for annotation information pertaining to a file of identified DNA variants from a patient’s tumor sample. *PredictProteinConsequence* predicted the potential molecular effect of the resultant amino acid changes caused by the DNA variant identified from a patient’s tumor sample. *MergeAnnotation* merged all annotation information obtained from the other components, in addition to information obtained from a file of manually curated annotations that detail sequence characteristics of the identified DNA variant (for example, within a homopolymer region); it then output a final file detailing the annotation information for the identified DNA variants.

Individual components were then created for each component-type. For example, the components *PredictProteinConsequenceVersion1* and *PredictProteinConsequenceVersion2* were created under the *PredictProteintConsequence* component-type and the component *QueryLocalCOSMICVersion1* was created under the *QueryLocalCOSMIC* component-type. Each component was defined with the following: 1) input datasets, 2) computational code, and 3) output datasets. For example, each *PredictProteinConsequence* component was defined with the following two input datasets: 1) Patient_Calledt_DNA_Variant_File and 2) Transcript_File and the output dataset Predicted_Protein_Consequence (Fig. 2b). Thus, datasets not classified as a Patient_Called_DNA_Variant_File or Transcript_File dataset would not be a valid input into the *PredictProteinConsequence* component. Similarly, any output from the *PredictProteinConsequence* component would be classified as a Predicted_Protein_Consequence dataset. The code needed for the analysis of this step was included to complete the creation of the component. This component utilizes the Bioconductor VariantAnnotation package [31] for its analysis (please refer to "Clinical Omics Analysis Pipeline" section for more detail); however, code implementing other popular annotation methods may easily be incorporated or used in its place. Please refer to Table 3 for a complete description of all input/output datasets for each component-type.

**Table 3:** WINGS input/output datasets for each component-type within our clinical omics use-case

Component-type	Input dataset(s)	Output dataset(s)
CreateLocalCOSMIC	GeneTrails_Genes	COSMICSubset
CreateLocalSNP	GeneTrails_Genes	SNPSubset
QueryLocalCOSMIC	Patient_Called_DNA_Variant_File, COSMICSubset	Queried_COSMIC_Result
QueryLocalSNP	Patient_Called_DNA_Variant_File, SNPSubset	Queried_SNP_Result
PredictProteinConsequence	Patient_Called_DNA_Variant_File, Transcript_File	Predicted_Protein_Consequence
MergeAnnotation	Pateint_Called_Variant_File, Queried_COSMIC_Result, Queried_SNP_Result, Predicted_Protein_Consequence, In_House_Curation_of_DNA_Variants	Final_Annotation_of_DNA_Variants

#### Semantic rules and constraints

Workflow rules and constraints can be used to enforce user-defined rules/constraints needed within a workflow template to create a semantically validated workflow run such as any pre-specified requirements for input datasets, inter-dependencies between components and/or datasets, or recommended/proposed regulations. Rules and constraints currently defined within our clinical workflow include requiring that genomic coordinates across all datasets be of the same genomic assembly and ensuring the propagation of pre-defined sets of metadata (e.g., patient ID number, software versions, data set versions) throughout a workflow run. Effective metadata propagations aid in effective provenance tracking. User-defined rules and constraints have also been put in place to pre-define the use of specific components, within each of our component-types, with specific versions of datasets. For example, a rule has been defined specifying that the UseComponentVersion metadata value in the Transcript_File dataset must be equal to the ComponentVersion parameter value of the *PredictProteinConsequence* component used. Every component under the *PredictProteinConsequence* component-type has a value set for ComponentVersion, indicating its version number, and set to match the value of the UseComponentVersion metadata value a Transcript_File dataset. Thus, a user is effectively choosing a specific component from a component type when choosing a specific input dataset. Similar rules have been set up for pre-defining the use of specific components within each component type. Please refer to the Additional file 1 for a full list of rules and constraints defined within our clinical omics workflow.

### Assembly of a workflow run

**Fig. 3:**
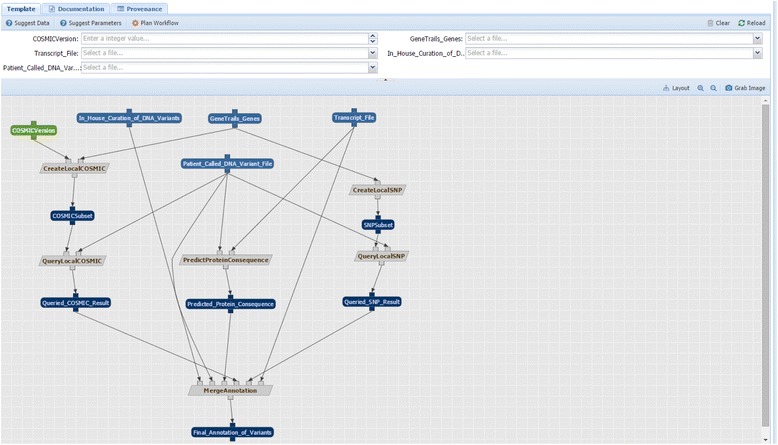
WINGS workflow template for our clinical omics use-case. WINGS templates are fully connected representations of all components, datasets, and rules and constraints of an analysis pipeline needed to execute a semantically validated workflow run. A workflow template representing our clinical omics analysis pipeline. Within our workflow template, each step is represented by its component-type (*grey rectangles*); however, please note that individual components can also be sequentially connected to one another to build a workflow template that has all input and output datasets (*blue rounded rectangles*) represented. Once a workflow template is created, WINGS generates an accompanied GUI for the workflow template, thus allowing workflow users to execute workflow runs. Due to the enforcement of all user-defined rules and constraints, each workflow run is semantically validated. Pre-defined rules and constraints also enables WINGS to help guide users through a workflow run by suggesting semantically validated inputs and parameters (*Suggest Data* and *Suggest Parameters* buttons). For example, due to our predefined rules and constraints, only datasets with the same genomic assembly would be suggested for this workflow template

Once all datasets, components, rules and constraints are defined and created, each can be pieced together to assemble a workflow template (Fig. 3). Our workflow template was assembled using only component-types; however, individual components can also be used to build a workflow template. The workflow template illustrates each step of our analysis pipeline in addition to all input and output datasets.

### Execution of a workflow run

Workflow users interact with WINGS in a different way from a workflow developer. Workflow users do not need to know how the workflow was developed in order to use it. Upon the creation of a workflow template, WINGS generates a GUI for workflow users to interact with and run assembled workflows (see top of Fig. 3). With this GUI, users are able to choose the desired parameters and inputs for this workflow. Furthermore, through the semantic reasoning [28, 32] of pre-defined rules and constraints, the ‘Suggest Parameters’ and ‘Suggest Data’ buttons within the GUI can be used to suggest appropriate parameters and inputs, respectively, for a workflow run. This guides users effectively and accurately through a workflow run. For example, due to our pre-defined rules and constraints, upon the selection of a Patient_Called_DNA_Variant_File, WINGS would only allow the selection of additional input objects of the same genomic assembly, as specified in their individual GenomicAssembly metadata. If a user chooses an input inconsistent with the pre-defined rules and constraints, a message is displayed informing the user of the error and requiring the user to choose an alternative input. Once all parameters and inputs are provided, the workflow run can be planned and ultimately run with the ‘Plan Workflow’ button. As the workflow run is being executed, WINGS directs users to a user interface where the run can be monitored and, when needed, reports from code execution failures are displayed to aid in debugging workflows and the underlying code.

### Execution of our clinical omics workflow

**Fig. 4:**
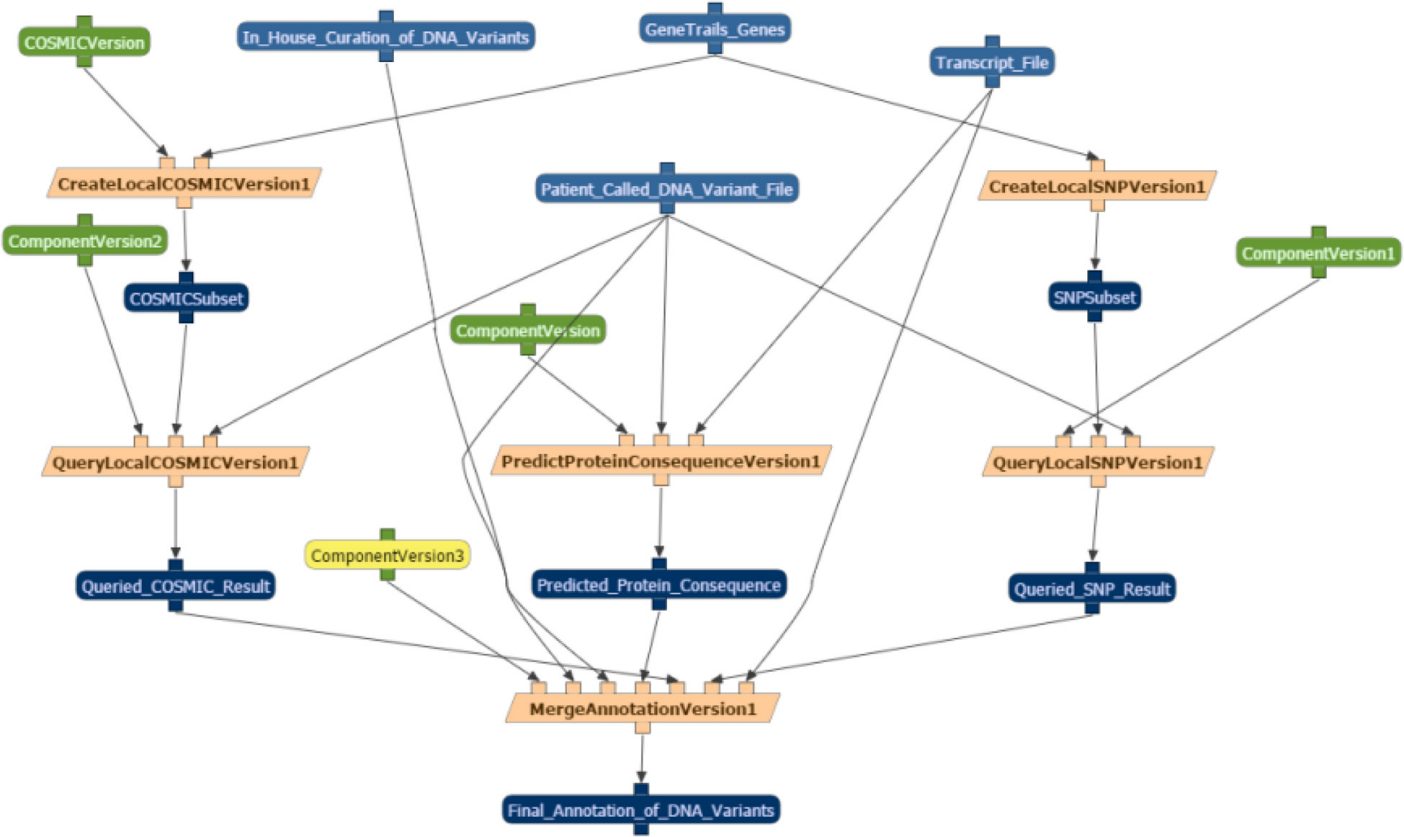
Execution of our clinical omics use-case WINGS workflow. Once a workflow run is executed, the details of the run are shown. Displayed is the successful execution of our clinical omics use-case WINGS workflow. All input parameters (*green*), input and output data objects (*blue*), and individual components (*yellow*) of the workflow run are shown. Particularly when component-types are used to define a workflow template, the details of an executed workflow run can be used to identify the exact components used for each workflow run. Based on the chosen input datasets and the user-defined rules and constraints, Version1 of each component-type was used in our executed workflow run

The executed workflow plan of a successful run of our clinical omics workflow highlighting all parameters, datasets, and components used is shown in Fig. 4. Particularly when component-types are used to assemble a workflow run, as in our clinical omics pipeline, this schema shows the actual components used during the execution as these may change as data inputs change. Based on the use of the same input data and versions of annotation sources, the final output from this workflow run was found to be identical (based on the use of the unix *diff* command) to the output obtained from the original analysis pipeline. Our final workflow output had the added benefits of having all run-time parameters and metadata automatically tracked and the assurance that all parameters, datasets, and components used during the analysis were consistent with all user-defined rules and constraints. Please refer to Additional file 1 for more detailed instructions on how to execute a run of our clinical omics workflow on the WINGS site.

### Meeting the minimal requirements of transparency and reproducibility of clinical omics

Based on the checklist of requirements recommended for enhanced transparency and reproducibility of translational and clinical omics defined in Table 1, our WINGS implemented clinical omics workflow met all requirements. All data, including the exact input data used, intermediate data, third party data, output data, and their provenance, were captured and preserved within our implemented workflow. All code, configurations, computing environment, and their provenance were preserved along with a high level diagram illustrating all steps of the analysis. And most importantly, the user-defined rules and constraints within our workflow provided the veracity checks needed to enhance analytical validity.

## Discussion

The implementation of our clinical omics DNA variant annotation pipeline use-case within the WINGS platform is the first implementation and execution of a clinical omics pipeline in a semantic workflow. We found that the implementation of our clinical omics annotation pipeline into a semantic workflow helped us to achieve the requirements for enhanced transparency, reproducibility, and analytical accuracy recommended for translational and clinical omics. During the implementation of our clinical omics workflow, we also found many features of the WINGS system were particularly primed to support the specific needs of clinical omics analyses. These include the need to: 1) keep pace with frequent updates of biological life science databases; 2) enforce consistency and data integrity across heterogeneous biological and clinical data; 3) keep pace with rapid updates and development of omics software tools; and 4) process large omics data sets. Each is described below.

### Frequent updates of molecular life science databases

The analysis and interpretation of omics data rely heavily on information within molecular life science databases such as those provided by the National Center for Biotechnology Information (NCBI) [57], European Molecular Biology Laboratory — European Bioinformatics Institute (EMBL-EBI) [58], and the UCSC Genome Browser [59]. Gene and transcript information supplied by NCBI’s Reference Sequence (RefSeq) database [60] and EMBL-EBI Ensembl database [61] serves as the foundation of many omics studies, particularly in RNA-seq studies [62]. Databases such as dbSNP, COSMIC, and clinVAR [63] provide annotation information for DNA variants regarding their frequency within the population and potential associations with disease and clinical phenotype.

To keep pace with our growing biological knowledge, information within these databases is constantly updated. For example, RefSeq databases are updated twice a month [60], the COSMIC database is updated every 2 months [64], and new builds of dbSNP are periodically released, especially after a new genome release or after a large submission of SNPs [30]. To ensure that the most current biological knowledge is used to analyze and interpret omics data, particularly within a clinical setting, it is imperative that all provenances of the databases are effectively captured and tracked.

WINGS’ ability to dynamically extract and propagate metadata within a component enhances the capture and tracking of provenance of datasets associated with frequently updated biological databases. The ability to dynamically extract metadata within a component is a new and unique feature of WINGS that helps to prevent any errors that may arise if manual intervention were needed. For example, the version of R used within each component of our clinical omics workflow is dynamically extracted at runtime and automatically propagated to the RVersionId metadata value of its output dataset. Within other workflow platforms, such as Galaxy and Taverna, metadata can only be manually populated and cannot be dynamically extracted at runtime.

### Heterogeneity/consistency of biological data

The analysis and interpretation of omics data also rely heavily on disparate and heterogeneous sets of biological data. For example, a typical RNA-seq analysis protocol involves two very different types of biological data: 1) the genomic sequence used for the alignment of the RNA-seq reads; and 2) the annotated transcript models used for expression quantification. Within our DNA variant annotation pipeline, biological information across multiple databases is used. Thus, to ensure consistency and validity across these heterogeneous data sources, it is critical that the disparate data types be consistent with one another.

The WINGS platform helps to ensure consistency across heterogeneous data sets through the use of its semantic technology. For our clinical omics workflow, user-defined rules and constraints were used to ensure that all datasets were of the same genomic assembly and that specific datasets were processed using specific workflow components. Further enhancing the consistency across disparate datasets is WINGS ability to predefine and constrain the specific datasets allowed as input/output for each component. Predefining and constraining the types of datasets helps to maintain the integrity of the datasets used. These features to enhance data integrity and veracity are absent in other workflow platforms.

### Rapid development of omics software tools

Paralleling, and at times even driven by, our growth of biological knowledge is the rapid development of new and existing omics analysis software tools. As an example, two popular short-read alignment tools, BWA [65] and TopHat [66], had a total of seven and three releases, respectively, in the year 2014. For a workflow system to effectively support clinical omics, in addition to efficiently tracking the specific versions of the software used, it is critical that the integration of new or updated software tools within new or existing workflows be user-friendly and efficient.

Two features of the WINGS platform help to efficiently incorporate new tools and updates to existing tools. The first feature is WINGS’ ability to group related components under a common component-type: this allows components for alternative tools or updated versions of existing tools to be easily added into an existing workflow template and their use semantically enforced. Related to this, the second feature is its ability to track the provenance of all component-types, components and workflow templates. A timestamp and user-ID is associated with the creation and update of each. Provenance for data objects is also similarly tracked.

### Processing of large omics data sets

The ability to store and process large data sets has become a mandatory part of analyzing omics data, particularly as the volume and complexity of omics data continue to increase [67, 68]. WINGS’ ability to execute workflows under a variety of modes — either in a local host, across a network of local machines, or across large scale distributed data processing environments, such as clusters or cloud services — is an invaluable tool in processing large omics data sets.

## Conclusions

We implemented and executed a clinical omics pipeline aimed at annotating DNA variants identified through large-scale DNA sequencing using the WINGS semantic workflow system. We found the semantic workflows in WINGS capable of effectively meeting the requirements for enhanced transparency, reproducibility, and analytical validity recommended for translational and clinical omics. We further found many features of the WINGS platform particularly effective in supporting the specific needs of clinical omics analyses.

The next stage for the application of WINGS in this setting is extension to other clinical omics use cases, as well as clinical user evaluation to facilitate seamless integration in these settings. We also note that the needs for reproducibility extend beyond the clinical setting. With regard to methods development, the semantic constraints in WINGS allow for more efficient and robust dissemination of methods and workflows to the broader research community, particularly to non-expert users. The FDA’s Computational Science Center has now started to receive next generation sequencing data with regulatory submissions that must be validated and analyzed, along with the corresponding methods. For FDA approval diagnostic devices, analytical validation of the device to establish performance characteristics, such as analytical specificity, precision (repeatability and reproducibility), and limits of detection, is essential. As such validation may require developing an algorithm or determining the threshold for clinical decisions, these steps must be captured such that the rationale and evidence for these decisions can also be evaluated. Finally, given the National Institutes of Health’s initiatives to improve reproducibility, particularly in preclinical research, frameworks such as WINGS will become more and more essential to the research enterprise.

## Additional file

Additional file 1:
**Dataset metadata.** (DOCX 20 kb)

## Abbreviations

COSMIC: Catalogue of Somatic Mutations in Cancer
dbSNP: Single Nucleotide Polymorphism database
EMBL-EBI: European Molecular Biology Laboratory — European Bioinformatics Institute
FDA: Food and Drug Administration
NCBI: National Center for Biotechnology Information
SNP: single-nucleotide polymorphism
WINGS: Workflow Instance Generation and Specialization
