# Supplementary material for: Use of semantic workflows to enhance transparency and reproducibility in clinical omics
Source: Genome Med. 2015 Jul 25;7(1):73. doi: 10.1186/s13073-015-0202-y (PMC4545705; doi:10.1186/s13073-015-0202-y)
Supplement: Additional file 1: — Dataset metadata. (DOCX 20 kb) [file 13073_2015_202_MOESM1_ESM.docx]

# Dataset Metadata

GeneTrails_Genes:

- version (int): version number
- source (string): data source (i.e., Hugo Gene Nomenclature Committee)

COSMICSubset:

- COSMICVersionId (int): version of COSMIC
- GenomicAssembly (string): genomic assembly of genomic coordinates
- UsedComponentVersion (int): version number of *CreateLocalCOSMICSubset* used
- UseComponentVersion (int): version number of *QueryLocalCOSMIC* to be used
- Versions of R and R packages used to create the data object (string)

SNPSubset:

- dbSNPVersionId (int): version of dbSNP
- GenomicAssembly (string): genomic assembly of genomic coordinates
- UsedComponentVersion (int): version number of *CreateLocalSNPSubset* used
- UseComponentVersion (int): version number of *QueryLocalSNP* to be used
- Versions of R and R packages used to create the data object (string)

Patient_Called_DNA_Variant_File:

- PatientId (int): unique identifier for patient sample
- GenomicAssembly (string): genomic assembly of genomic coordinates

Queried_COSMIC_Result:

- PatientId (int): unique identifier for patient sample
- COSMICVersionId: version COSMIC
- GenomicAssembly (string): genomic assembly of genomic coordinates
- UsedComponentVersion (int): version number of *QueryLocalCOSMIC* used
- Versions of R and R packages used to create the data object (string)

Queried_SNP_Result:

- PatientId (int): unique identifier for patient sample
- dbSNPVersionId: version of dbSNP
- GenomicAssembly (string): genomic assembly of genomic coordinates
- UsedComponentVersion (int): version number of *QueryLocalCOSMIC* used
- Versions of R and R packages used to create the data object (string)

Transcript_File:

- TranscriptSource (string): data source (i.e., NCBI RefSeq)
- TranscriptFileVersionId (int): version of transcript_file
- GenomicAssembly (string): genomic assembly of genomic coordinates
- UseComponentVersion (int): version of *PredictProteinConsequence* to be used

Predicted_Protein_Consequence:

- PatientId (int): unique identifier for patient sample
- GenomicAssembly (string): genomic assembly of genomic coordinates
- UsedComponentVersion (int): version number of *PredictProteinConsequence* used
- TranscriptFileVersionId (int): version of transcript_file used
- Versions of R and R packages used to create the data object (string)

In_House_Curation_of_DNA_Variants:

- InHouseCurationVersionId (int): version of In_House_Curation_of_DNA_Variants
- GenomicAssembly (string): genomic assembly of genomic coordinates
- UseComponent (int): version of *MergeAnnotation* to be used

Final_Annotation_of_DNA_Variants:

- PatientId (int): unique identifier for patient sample
- GenomicAssembly (string): genomic assembly of genomic coordinates
- UsedComponentVersion (int): version number of *MergeAnnotation* used
- Versions of R and R packages used to create the data object (string)

# Semantic Rules and Constraints

Rules and constraints were encapsulated within individual workflow component-types/components. Below is a list of all semantic rules and constraints.

*CreateLocalCOSMIC:* 1) Have the version of COSMIC be propagated to the COSMICVersionId metadata for the COSMICSubset data-object; 2) Obtain and propagate the version of R and R packages onto their respective metadata values for the COSMICSubset data-object.

*CreateLocalSNP*: 1) Have the version of dbSNP be propagated to the dbSNPVersionId metadata for the SNPSubset data-object; 2) Obtain and propagate the version of R and R packages onto their respective metadata values for the SNPSubset data-object.

*QueryLocalCOSMIC*: 1) The GenomicAssembly metadata of the Local_COSMIC_subset data-object must be the same as the GenomicAssembly metadata of the Patient_Called_DNA_Variant_File data-object; 2) Have the PatientId metadata from the Patient_Called_DNA_Variant_File data-object be propagated to the PatiendId metadata for the Queried_COSMIC_Result data-object; 3) Have the GenomicAssembly metadata from the Patient_Called_DNA_Variant_File data-object be propagated to the GenomicAssemly metadata for the Queried_COSMIC_Result data-object; 4) Have the UseComponentVersion metadata for the Local_COSMIC_Subset data-object be the same as the ComponentVersion parameter of the *QueryLocalCOSMIC* component; 5) Have the ComponentVersion parameter of the *QueryLocalCOSMIC* component used be propagated to the UsedComponentVersion metadata of the Query_COSMIC_Result data-object; 6) Obtain and propagate the version of R and R packages onto their respective metadata values for the Queried_COSMIC_Result data-object.

*QueryLocalSNP*: 1) The GenomicAssembly metadata of the Local_SNP_subset data-object must be the same as the GenomicAssembly metadata of the Patient_Called_DNA_Variant_File data-object; 2) Have the PatientId metadata from the Patient_Called_DNA_Variant_File data-object be propagated to the PatiendId metadata for the Queried_SNP_Result data-object; 3) Have the GenomicAssembly metadata from the Patient_Called_DNA_Variant_File data-object be propagated to the GenomicAssemly metadata for the Queried_SNP_Result data-object; 4) Have the UseComponentVersion metadata for the Local_SNP_Subset data-object be the same as the ComponentVersion parameter of the *QueryLocalSN*P component; 5) Have the ComponentVersion parameter of the *QueryLocalSNP* component used be propagated to the UsedComponentVersion metadata of the Query_SNP_Result data-object; 6) Obtain and propagate the version of R and R packages onto their respective metadata values for the Queried_SNP_Result data-object.

*PredictProteinConsequence*: 1) The GenomicAssembly metadata of the Transcript_File data-object must be the same as the GenomicAssembly metadata of the Patient_Called_DNA_Variant_File data-object; 2) Have the PatientId metadata from the Patient_Called_DNA_Variant_File data-object be propagated to the PatiendId metadata for the Predicted_Protein_Consequence data-object; 3) Have the GenomicAssembly metadata from the Patient_Called_DNA_Variant_File data-object be propagated to the GenomicAssemly metadata for the Predicted_Protein_Consequence data-object; 4) Have the UseComponentVersion metadata for the Transcript_File data-object be the same as the ComponentVersion parameter of the *PredictProteinConsequence* component; 5) Have the ComponentVersion parameter of the *PredictProteinConsequence* component used be propagated to the UsedComponentVersion metadata of the Predicted_Protein_Consequence data-object; 6) Obtain and propagate the version of R and R packages onto their respective metadata values for the Predicted_Protein_Consequence data-object.

*MergeAnnotation*: 1) The GenomicAssembly metadata of the In_House_Curation_of_DNA_Variants data-object must be the same as the GenomicAssembly metadata of the Patient_Called_DNA_Variant_File data-object; 2) Have the PatientId metadata from the Patient_Called_DNA_Variant_File data-object be propagated to the PatiendId metadata for the Final_Annotation_of_DNA_Variants data-object; 3) Have the GenomicAssembly metadata from the Patient_Called_DNA_Variant_File data-object be propagated to the GenomicAssemly metadata for the Final_Annotation_of_DNA_Variants data-object; 4) Have the UseComponentVersion metadata for the In_House_Curation_of_DNA_Variants data-object be the same as the ComponentVersion parameter of the *MergeAnnotation* component; 5) Have the ComponentVersion parameter of the *MergeAnnotation* component used be propagated to the UsedComponentVersion metadata of the Final_Annotation_of_DNA_Variants data-object; 6) Obtain and propagate the version of R and R packages onto their respective metadata values for the Final_Annotation_of_DNA_Variants data-object.

# Running our Clinical Omics Workflow on the WINGS Public Site

Please following the instructions below to access and run the clinical omics workflow described in this manuscript.

Use the following link to access our workflow:

[http://www.wings-workflows.org/wings-portal/users/genmed/ClinicalOmics/workflows](https://mail.ohsu.edu/owa/redir.aspx?SURL=4--w-TB7ZPfeVkQguYh9RJ9JLZH2iwpwAzYR9tG3Ey6S0H7d5X3SCGgAdAB0AHAAOgAvAC8AdwB3AHcALgB3AGkAbgBnAHMALQB3AG8AcgBrAGYAbABvAHcAcwAuAG8AcgBnAC8AdwBpAG4AZwBzAC0AcABvAHIAdABhAGwALwB1AHMAZQByAHMALwBnAGUAbgBtAGUAZAAvAEMAbABpAG4AaQBjAGEAbABPAG0AaQBjAHMALwB3AG8AcgBrAGYAbABvAHcAcwA.&URL=http%3a%2f%2fwww.wings-workflows.org%2fwings-portal%2fusers%2fgenmed%2fClinicalOmics%2fworkflows)

with the following credentials:

Username: genmed

Password: genmed123

After logging onto the portal, you will be directed to the ‘VariantAnnotation’ workflow template which is the genomic annotation portion of the workflow described in our manuscript.  For simplicity and access requirements (i.e., credentials are needed to download COSMIC data), we have pre-run the ‘CreateLocalCOSMIC’ and ‘CreateLocalSNP’ components thus the ‘COSMICSubset’ and ‘SNPSubset’ data sets are available for use with the workflow.  To execute a run of the workflow, the appropriate data sets must be provided.  With the semantic enforcement of pre-defined rules and constraints, WINGS helps guide users to the use of the appropriate data sets with 1) the use the ‘SuggestData’ feature wherein set(s) of semantically validated data sets are provided or 2) the use of individual pull drop down menus.  Error messages warn users of the use of inconsistent/incorrect data sets.  Once all data sets have been chosen, the ‘Plan Workflow’ button can be used to detail the exact components to be used during the workflow run.  This is particularly informative in cases where workflow templates are built using component types (as exemplified by our current workflow template).  When a workflow is selected, the details of workflow run will be displayed.  Users can then run the workflow by pressing the ‘Run Selected Workflow’ button.  A window will then pop up directing users to the ‘Access Runs’ page to monitor the execution.  Users can return to this page at any time by selecting the ‘Access Run’ tab under the ‘Analysis’ tab on the top of the page.  The ‘Access Runs’ page can also be used to access and save data objects generated during a workflow run.  Users can view or save any generated data objects.  Saved data objects can be viewed using the ‘Manage Data’ tab under the ‘Advanced’ tab on the top of the page.  To execute additional runs of this workflow template, please access the ‘Run Workflows’ tab under the ‘Analysis’ tab on the top of the page.  For more detailed information on individual components and/or data sets, please access their respective tabs under the ‘Advanced’ tab on the top of the page.

For a more detailed tutorial on the WINGS system, please see the following [http://www.wings-workflows.org/tutorial](https://mail.ohsu.edu/owa/redir.aspx?SURL=HmO87FahhyQKADYXft8PxbTq30ysfvMPiswq11g7yT6S0H7d5X3SCGgAdAB0AHAAOgAvAC8AdwB3AHcALgB3AGkAbgBnAHMALQB3AG8AcgBrAGYAbABvAHcAcwAuAG8AcgBnAC8AdAB1AHQAbwByAGkAYQBsAA..&URL=http%3a%2f%2fwww.wings-workflows.org%2ftutorial)
